# A systematic review and meta-analysis of blood level of MCP-1/CCL-2 in severe and uncomplicated malaria

**DOI:** 10.1038/s41598-024-80201-y

**Published:** 2024-11-20

**Authors:** Manas Kotepui, Pattamaporn Kwankaew, Aongart Mahittikorn, Kwuntida Uthaisar Kotepui, Frederick Ramirez Masangkay, Apichai Wattanapisit, Polrat Wilairatana

**Affiliations:** 1 Medical Technology Program, Faculty of Science, Nakhon Phanom University, Nakhon Phanom, Thailand; 2 Medical Technology, School of Allied Health Sciences, Walailak University, Tha Sala, Nakhon Si Thammarat, Thailand; 3 Department of Protozoology, Faculty of Tropical Medicine, Mahidol University, Bangkok, 10400 Thailand; 4 Department of Medical Technology, Faculty of Pharmacy, University of Santo Tomas, Manila, 1008 Philippines; 5 School of Medicine, Walailak University, Tha Sala, Nakhon Si Thammarat, Thailand; 6 Department of Clinical Tropical Medicine, Faculty of Tropical Medicine, Mahidol University, Bangkok, 10400 Thailand

**Keywords:** MCP-1, CCL-2, Malaria, *Plasmodium*, Systematic review, Meta-analysis, Chemokines, Malaria

## Abstract

Monocyte chemoattractant protein-1 (MCP-1) or C-C Motif Chemokine Ligand 2 (CCL-2) is a key chemokine implicated in the inflammatory response to malaria. The objective of the study was to synthesize the evidence on variations in MCP-1/CCL-2 levels in relation to *Plasmodium* spp. infections and the severity of malaria. The protocol for this systematic review and meta-analysis was registered at PROSPERO (CRD42024565867). To identify relevant studies, a comprehensive search strategy was conducted using major databases, including PubMed, Scopus, Embase, Medline, Journals@Ovid, and Nursing & Allied Health Premium. The Joanna Briggs Institute (JBI) critical appraisal checklists were used to determine the risk of bias in selected studies. The pooled standardized mean difference (SMD, Hedge’s g) and their 95% confidence interval (CI) were estimated using the random-effects model. Thirty-three studies, with a total of 6,804 participants enrolled, were included in the systematic review. Most studies (60.6%) were published between 2010 and 2019; the majority (57.6%) were conducted in Africa. The predominant *Plasmodium* species studied was *P. falciparum* (66.7%). The meta-analysis revealed no significant difference in MCP-1/CCL-2 levels between *Plasmodium*-infected and uninfected individuals (*P*: 0.16, SMD: 0.99, 95% CI: -0.39–2.37, *I*^*2*^: 97.2%, number of participants: 2140). Subgroup analysis found an increase in MCP-1/CCL-2 levels in children with *Plasmodium* infections, with no heterogeneity observed (*I*^*2*^: 0%). Subgroup analysis found no difference in MCP-1/CCL-2 levels between *P. falciparum*-infected participants and uninfected participants, as well as between *P. falciparum* or non-*P. falciparum*-infected participants and uninfected participants. The meta-analysis revealed significantly higher MCP-1/CCL-2 levels in participants with severe *Plasmodium* infections compared to those with non-severe malaria (*P*: 0.04, SMD: 1.51, 95% CI: 0.06–2.95, *I*^*2*^: 98.5%, number of participants: 1371). The systematic review and meta-analysis suggest no statistically significant difference in MCP-1/CCL-2 levels in participants with *Plasmodium* infections overall. However, there was a significant increase in MCP-1/CCL-2 levels in patients with severe malaria. These findings suggest that MCP-1/CCL-2 may have potential as a prognostic biomarker for severe malaria. Future research should focus on large-scale, well-designed studies to validate the role of MCP-1/CCL-2 in malaria and further explore its prognostic potential.

**Supplementary Information:**

The online version contains supplementary material available at 10.1038/s41598-024-80201-y.

## Introduction

Malaria, caused by *Plasmodium* spp. and transmitted through female *Anopheles* mosquitoes, remains a major global health concern, particularly in tropical and subtropical regions^1,2^. In 2022, an estimated 249 million cases were reported across 85 endemic countries, with most cases occurring in these regions^1^. *Plasmodium falciparum* and *Plasmodium vivax* are responsible for the majority of infections, while other species, including *Plasmodium malariae*, *Plasmodium ovale wallikeri*, *Plasmodium ovale curtisi*, and *Plasmodium knowlesi*, contribute to the remaining cases^3^. Additionally, asymptomatic *Plasmodium* infections, which do not present typical symptoms, complicate efforts for malaria elimination^4^.

The immune response to malaria involves both innate and adaptive immunity^5,6^. Upon *Plasmodium* infection, innate immune cells such as macrophages, dendritic cells, and natural killer (NK) cells are activated, leading to the production of pro-inflammatory cytokines, including interleukin-6 (IL-6), tumor necrosis factor-alpha (TNF-α), and interferon-gamma (IFN-γ), which help control the infection and activate adaptive immune responses^7^. Chemokines play a critical role in coordinating the host’s defense mechanisms by regulating white blood cell migration^8–10^. Chemokine (C-C motif) ligand 2 (CCL2), also known as monocyte chemoattractant protein-1 (MCP-1), is a key chemokine involved in recruiting monocytes, memory T cells, dendritic cells, and NK cells to sites of tissue injury or infection^10,11^. Various cell types produce MCP-1/CCL-2 in response to inflammatory stimuli and bind to its receptor, CCR2, to promote cell migration and activation^12–17^. It also plays a role in regulating inflammatory responses and cancer development^18^.

In the context of malaria, MCP-1/CCL-2 modulates the immune response to *Plasmodium* infections, particularly through its involvement in cerebral malaria^8^. The pre-clinical study demonstrated that CCR2 expression was significantly upregulated by CD8 + T cells isolated from the brain and spleen of malaria-infected mice, suggesting that trafficking through these chemokine receptors may be involved in recruiting inflammatory cells during severe malaria^8,19^. Additionally, mice lacking CCR2 were completely susceptible to cerebral malaria, highlighting the importance of MCP-1/CCL-2 in malaria pathophysiology. However, while these findings provide valuable insights, it is important to note that preclinical studies may not fully translate to human malaria, and further research is needed to clarify MCP-1/CCL-2’s role in human infection. The objective of this study was to synthesize evidence on variations in MCP-1/CCL-2 levels in relation to *Plasmodium* infections and malaria severity. A systematic review and meta-analysis approach was employed to provide a robust assessment of MCP-1/CCL-2’s potential as a biomarker for malaria severity. The study aimed to offer insights into how MCP-1/CCL-2 contributes to malaria pathophysiology, with implications for diagnostic and therapeutic development.

## Methods

### Registration protocol and guidelines for reporting

The systematic review protocol was registered in PROSPERO (registration number: CRD42024565867). The systematic review followed the Preferred Reporting Items for Systematic Reviews and Meta-Analyses (PRISMA) guidelines^20^.

### Research question framework

The research question followed the Population, Exposure, Comparator, Outcome (PECO) guideline^21^. In this context, ‘P’ represents participants diagnosed with *Plasmodium* infections; ‘E’ denotes the presence of *Plasmodium* infection; ‘C’ includes *Plasmodium*-uninfected individuals or those with non-severe types of malaria; and ‘O’ indicates the measurement of circulating MCP-1/CCL-2 levels in the participants’ blood (serum or plasma).

### Definitions

Severe malaria refers to the presence of asexual parasites in the peripheral blood with clinical or laboratory evidence of vital organ dysfunction, as defined by the World Health Organization’s criteria for severe malaria^22^. Non-severe types of malaria include participants infected with *Plasmodium* species who do not exhibit clinical or laboratory evidence of vital organ dysfunction, such as those with uncomplicated (mild) malaria. Asymptomatic infections are characterized by the presence of asexual parasites in the peripheral blood without accompanying clinical symptoms^23^.

### Search strategy

To identify relevant studies, a comprehensive search strategy was conducted using major databases, including PubMed, Scopus, Embase, Medline, Journals@Ovid, and Nursing & Allied Health Premium. Keywords used in the search strategy included “CCL2”, “MCP-1”, and “malaria”, combined using Boolean operators “AND” or “OR” as follows: (“Chemokine CCL2” OR CCL2 OR “Monocyte Chemotactic and Activating Factor” OR “Monocyte Chemoattractant Protein-1” OR “Monocyte Chemoattractant Protein 1” OR “Chemokine (C-C Motif) Ligand 2” OR “CCL2 Chemokine” OR CCL2 OR “Monocyte Chemotactic Protein-1” OR “Monocyte Chemotactic Protein 1” OR MCP-1) AND (malaria OR *Plasmodium* OR “*Plasmodium* Infection“ OR “Remittent Fever“ OR “Marsh Fever“ OR Paludism). Medical Subject Headings (MeSH terms) were used in the PubMed search to maximize retrieval of relevant literature, while Emtree terms were utilized in the Embase search (Table S1). Additionally, searches were conducted in Google Scholar and through the reference lists of included studies to ensure comprehensive coverage of gray literature and relevant publications. There were no restrictions on the publication dates of the articles.

### Eligibility criteria

The eligibility criteria for this study were designed based on the PECO framework to ensure clarity and consistency. The *Population* includes participants diagnosed with *Plasmodium* infections, covering any species of the parasite. The *Exposure* refers to the presence of *Plasmodium* infection, regardless of severity. The *Comparator* involves *Plasmodium*-uninfected individuals or those with non-severe malaria, providing a basis for comparison. The *Outcome* focuses on measuring MCP-1/CCL-2 levels in the participants’ blood (either serum or plasma), which could be presented qualitatively or quantitatively. Exclusion criteria encompassed non-original articles such as case reports, case series, reviews, systematic reviews, conference abstracts, or letters to the editor. Additionally, studies involving non-human subjects, such as animal studies, in vitro studies, or computational models, were excluded. There were no restrictions on the study design of relevant articles, nor were there any restrictions based on language. The full texts of selected studies must be freely available in the publisher as open access. If not, the full texts of selected studies were requested directly from the author via e-mail or ResearchGate.

### Screening and selection

Articles from databases were managed using Endnote (version 20.0, Clarivate, USA). First, duplicates were removed before article screening. Second, the remaining articles were screened based on their relevant titles and abstracts. Third, after non-related articles were excluded, the full texts of the remaining articles were examined using predefined inclusion and exclusion criteria to identify studies that met the criteria. Finally, articles that met the criteria were included for critical appraisal and synthesis. Screening and selection of relevant articles were conducted by two independent authors (MK, AM). Any disagreements were resolved through discussion to achieve consensus.

### Data extraction and assessment of methodological quality

Data extraction was conducted using a standardized form to systematically collect relevant information from each included study. Extracted data included study characteristics (e.g., author, year, study design), participant characteristics (e.g., number, age ranges), exposures (*Plasmodium* infections), outcomes measured (MCP-1/CCL-2 in both qualitative and quantitative data in mean/standard deviation, median/range), as well as methods for *Plasmodium* detection and MCP-1/CCL-2 quantification. Data extraction was conducted by one author (MK) and fully cross-checked by another author (AM).

The Joanna Briggs Institute (JBI) critical appraisal checklists were used to determine the risk of bias in selected studies^24^. The criteria for assessing cross-sectional studies include clarity of inclusion criteria, detailed description of subjects and settings, validity and reliability of exposure and outcome measurements, and appropriateness of statistical analysis. The JBI critical appraisal checklist for cohort studies includes ensuring a similarity of exposed and unexposed groups at baseline, consistency in exposure measurement methods, validity and reliability of both exposure and outcome assessments, identification and consideration of potential confounding factors, and adequate follow-up duration with complete follow-up of participants. The JBI checklist for case-control studies evaluates ten criteria to ensure study quality: the similarity and appropriate matching of case and control groups, consistent identification criteria, standardized and reliable measurement of exposure and outcomes, identification and control of confounding factors, adequate exposure duration, and suitable statistical analysis. Two independent authors (MK, KUK) assessed methodological quality. Any disagreements were resolved through discussion to achieve consensus.

### Data synthesis

The SPICE framework (Setting, Perspective, Phenomenon of Interest, Comparison, Evaluation) was applied for thematic synthesis^25^. The *Setting* was malaria-endemic areas, the *Perspective* was participants with *Plasmodium* infections or severe malaria, the *Phenomenon of Interest* was MCP-1/CCL-2 levels, and the *Comparison* was uninfected participants or those with non-severe malaria. The pooled standardized mean difference (SMD) and their 95% confidence interval (CI) of MCP-1/CCL-2 between participants infected with *Plasmodium* species and those without the infections (non-malarial controls or healthy controls) was analyzed as the primary outcome of the meta-analysis. The SMD of MCP-1/CCL-2 between patients with severe and non-severe malaria was also analyzed as a secondary outcome. The SMD was pooled using the random-effects model described by DerSimonian^26^. SMD values less than 0.40 indicate small intervention effects, SMD values between 0.40 and 0.70 indicate moderate effects, and SMD values greater than 0.70 indicate large intervention effects, as previously described^27^. Heterogeneity was assessed using the *I²* statistic, with an *I²* value greater than 50% indicating significant heterogeneity^28^. Potential sources of heterogeneity among studies, such as differences in study characteristics, participant characteristics, or outcomes, were assessed and explored using meta-regression analysis and/or subgroup analyses^29^. Sensitivity analyses were performed to determine the stability of the meta-analysis results by omitting one study at a time^30^, and using different statistical models. Publication bias was determined by visualizing funnel plot asymmetry and using Egger’s test for meta-analyses that included 10 or more studies^31^. Statistical analyses were conducted using RStudio (Version: 2024.04.2 + 764)^32^.

## Results

### Search results

**Fig. 1:**
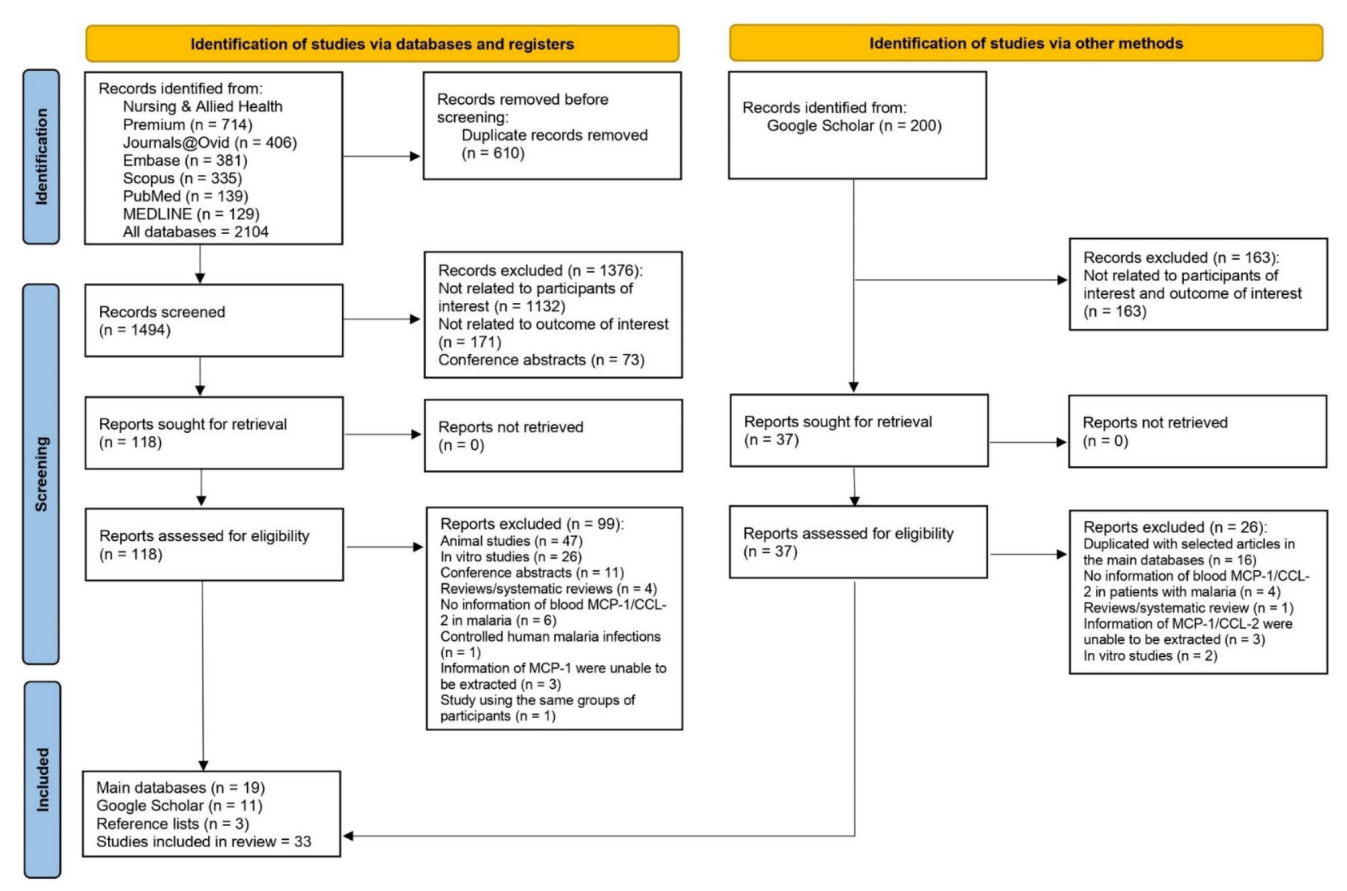
The PRISMA 2020 flow diagram illustrates the selection of studies for a systematic review of blood MCP-1/CCL-2 in malaria.

Figure 1 is the PRISMA 2020 flow diagram illustrating the selection process of the studies for a systematic review of blood MCP-1/CCL-2 in malaria. Initially, 2104 records were identified from six databases. After removing 610 duplicates, 1494 records were screened, and resulted in the exclusion of 1376 records for irrelevance. This left 118 records for detailed review, of which 99 were excluded for various reasons, such as animal or in vitro studies or lacking necessary MCP-1/CCL-2 information. Of 200 records from Google Scholar, 189 records were excluded during the screening processes. Finally, 33 studies were included in the review: 19 from main databases, 11 from Google Scholar, and three from reference lists.

### Summary characteristics of included studies

Thirty-three studies enrolled a total of 6804 participants (Table S2). Most studies (60.6%) were published between 2010 and 2019, with fewer studies from 2000 to 2009 (24.2%) and 2020 to 2023 (15.2%). The study designs included case-control studies (45.5%), cohort studies (39.4%), and cross-sectional studies (15.1%). Geographically, most studies were conducted in Africa (57.6%), particularly in countries like Ghana, Kenya, and Benin, while other studies took place in America (21.2%) and Asia (18.2%). The predominant *Plasmodium* species studied was *P. falciparum* (66.7%). Most studies (42.2%) enrolled severe and uncomplicated malaria patients. Participants were mainly children (33.3%), with significant portions of studies involving adults (21.2%), pregnant women (21.2%), and all age ranges (21.2%). *Plasmodium* detection in participants’ blood was primarily done through microscopy (54.6%), with some studies using PCR and RDT methods. MCP-1/CCL-2 levels were mostly measured using bead-based assays (81.8%), with plasma being the common sample type (87.9%) (Table 1).

**Table 1:** Summary characteristics of included studies.

Characteristics	*n* (33 studies)	%
Publication years		
2010–2019	20	60.6
2000–2009	8	24.2
2020–2023	5	15.2
Study design		
Case-control study	15	45.5
Cohort study	13	39.4
Cross-sectional study	5	15.1
Study area		
Africa	19	57.6
Ghana	3	9.1
Kenya	2	6.1
Benin	2	6.1
Malawi	2	6.1
Mali	2	6.1
Tanzania	2	6.1
Uganda	2	6.1
Mozambique	1	3.0
Senegal	1	3.0
Gabon	1	3.0
Cameroon	1	3.0
America	7	21.2
Brazil	5	15.2
Canada	1	3.0
Colombia	1	3.0
Asia	6	18.2
India	3	9.1
Myanmar	1	3.0
Malaysia	1	3.0
Thai-Myanmar border	1	3.0
Multi-continent (America/Asia/Oceania)	1	3.0
Brazil, Colombia, Guatemala, India, Papua New Guinea	1	3.0
*Plasmodium* species		
*P. falciparum*	22	66.7
Non-*P. falciparum*	5	15.1
*P. falciparum*, non-*P. falciparum*	6	18.2
Type of malaria		
Severe malaria/uncomplicated malaria	14	42.2
Uncomplicated malaria	4	12.1
Severe malaria	3	9.1
Symptomatic malaria*	2	6.0
Symptomatic malaria*/asymptomatic infections	2	6.0
Severe malaria/symptomatic malaria*/asymptomatic infections	1	3.0
Not specified	7	21.2
Participants		
Children	11	33.3
Adults	7	21.2
Pregnant women	7	21.2
All age ranges	7	21.2
Not specified	1	3.0
Methods for malaria detection		
Microscopic method	18	54.6
Microscopic method, PCR	7	21.2
Microscopic method/RDT	2	6.1
Microscopic method/RDT/PCR	5	15.1
Not specified	1	3.0
Methods for MCP-1/CCL-2 measurement		
Bead-based assays	27	81.8
ELISA	6	18.2
Blood samples		
Plasma	29	87.9
Serum	3	9.1
Unclear	1	3.0

*The authors did not specify whether the symptomatic malaria was severe or uncomplicated.

CCL-2, chemokine (C–C motif) ligand 2; ELISA, Enzyme-linked immunosorbent assay; MCP-1, Monocyte chemoattractant protein-1; PCR, polymerase chain reaction; RDT, rapid diagnostic test.

### Methodological quality of included studies

For cross-sectional studies, most studies met the criteria. However, two studies^33,34^ did not clearly identify or address confounding factors, indicating a potential risk of bias (Table S3). For case-control studies, while most met the key criteria such as comparability of groups, appropriate matching of cases and controls, standard criteria for identification, consistent and reliable measurement of exposure and outcomes, and appropriate statistical analysis, many did not clearly identify or address confounding factors. For cohort studies, most studies demonstrated strength in recruiting similar groups, valid exposure and outcome measurements, and using appropriate statistical methods. However, some studies show weaknesses in identifying confounding factors, addressing incomplete follow-up, and reporting reasons for loss of follow-up^35–43^.

### MCP-1/CCL-2 in participants with *Plasmodium* infections

The 24 studies explored MCP-1/CCL-2 levels in participants infected with *Plasmodium* species compared to those without infection^35,36,38,41–61^. Five studies focused on children, with mixed results: two studies found significantly higher MCP-1/CCL-2 levels in malaria patients compared to controls^38,54^, while two found no difference^45,47^, and one found higher levels only in severe cases^61^. Five studies on adults showed similar variations: three reported significantly higher MCP-1/CCL-2 levels in infected patients compared to healthy or uninfected controls^46,51,57^, while one found no difference^41^, and another reported significantly lower MCP-1/CCL-2 levels in infected patients^50^.

Studies spanning various age groups^43,53,55,56,59,60^ also showed diverse outcomes. Two studies reported higher MCP-1 levels in malaria patients^53,55^, while one found significantly higher MCP-1 levels in severe malaria compared to healthy controls but no difference between non-severe malaria and healthy controls^43^. The remaining studies found no significant differences^56,59,60^. In studies involving pregnant women, results were inconsistent, showing higher, lower, or no difference in MCP-1 levels among infected participants depending on the number of pregnancies (gravidity) and the type of blood sample taken (peripheral blood, cord blood, or placental samples)^35,36,42,44,48,52^.

Seven studies reported quantitative data on MCP-1/CCL-2 levels in *Plasmodium*-infected and uninfected individuals^36,38,44,46,48,49,60^. These studies were included in a meta-analysis to determine the pooled SMD of MCP-1/CCL-2 between the two participant groups. The results revealed no significant difference in MCP-1/CCL-2 levels between *Plasmodium*-infected and uninfected individuals (*P*: 0.16, SMD: 0.99, 95% CI: -0.39–2.37, *I*^*2*^: 97.2%, number of participants: 2140, Fig. 2).

**Fig. 2:**
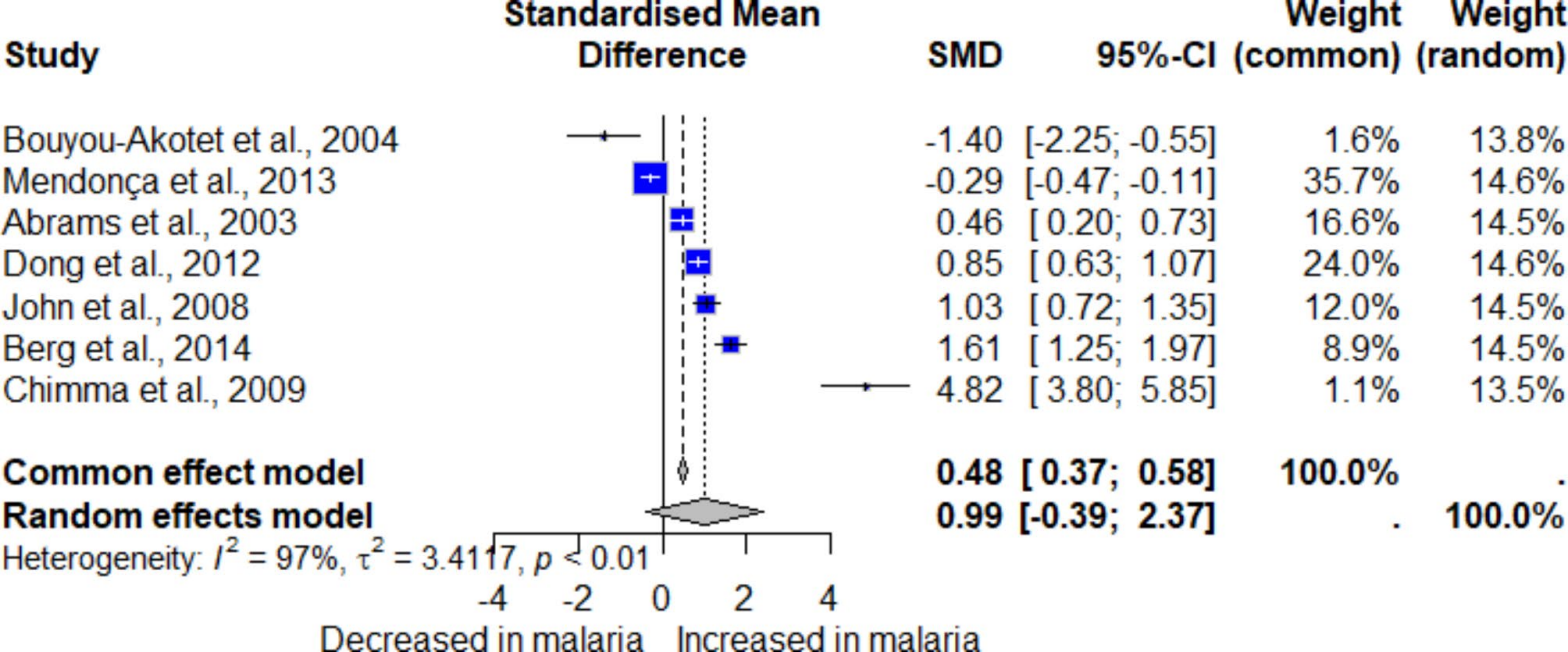
The forest plot illustrates the meta-analysis of MCP-1/CCL-2 levels between *Plasmodium*-infected and uninfected individuals. SMD represents the standardized mean differences. The CI illustrates the 95% confidence intervals from each study. The *I²* value of 97% reflects high heterogeneity across studies. The blue boxes illustrate an effect estimate from each study. The gray diamonds illustrate the pooled overall effect size.

The cumulative meta-analysis suggests a consistent trend toward no difference in MCP-1/CCL-2 levels between *Plasmodium*-infected and uninfected individuals (Supplementary Fig. 1). The subgroup meta-analysis of MCP-1/CCL-2 levels reveals considerable variability influenced by several factors (Table S4). Studies from different continents, participant groups, *Plasmodium* species, and diagnostic methods for malaria show significant differences in MCP-1/CCL-2 levels. The Asian study indicated higher MCP-1/CCL-2 levels, while African studies showed no difference, and the American study reported lower MCP-1/CCL-2 levels. Regarding participants, studies involving pregnant women demonstrated no difference in MCP-1/CCL-2 levels, whereas those enrolling only children or adults showed higher MCP-1/CCL-2 levels. For studies enrolling participants infected with *P. falciparum* only, no difference in MCP-1/CCL-2 levels between groups was observed. However, studies enrolling both *P. falciparum* and non-*P. falciparum* participants suggested higher MCP-1/CCL-2 levels, while the study focusing on non-*P. falciparum* infections alone demonstrated lower MCP-1/CCL-2 levels. Lastly, diagnostic methods contributed to the variability, with studies using microscopy alone generally showing no difference in MCP-1/CCL-2 levels between the two participant groups.

### MCP-1/CCL-2 in participants with severe *Plasmodium* infections

Ten studies investigated MCP-1/CCL-2 levels in severe versus uncomplicated malaria^33,38,39,43,56,59–63^. Herbert et al., found no difference in MCP-1 levels between severe non-cerebral malaria/multiorgan dysfunction and mild malaria. However, they observed significantly higher MCP-1 levels in cerebral malaria with multiorgan dysfunction compared to mild malaria. Additionally, no difference was found in MCP-1 levels between severe non-cerebral malaria and multiorgan dysfunction^56^. Dalko et al.^33^ found no difference in MCP-1/CCL-2 levels between cerebral malaria and mild malaria but observed significantly increased MCP-1/CCL-2 levels in acute renal failure compared to mild malaria. Lopera-Mesa et al.^39^ observed significantly higher MCP-1/CCL-2 levels in severe malaria (both cerebral and non-cerebral) compared to uncomplicated malaria. Tovar Acero et al.^43^ reported significantly higher MCP-1/CCL-2 levels in severe malaria compared to non-severe malaria. Jain et al.^59^ observed no difference in MCP-1/CCL-2 levels between cerebral malaria (survivors or non-survivors) and mild malaria. John et al.^38^ revealed no difference in MCP-1/CCL-2 levels between cerebral malaria and uncomplicated malaria. Mendonça et al.^60^ found no difference in MCP-1/CCL-2 levels between severe malaria (severe non-lethal and severe lethal malaria) and symptomatic/asymptomatic malaria. Ong’echa et al.^61^ reported no difference in MCP-1/CCL-2 levels between severe malarial anemia, non-severe malarial anemia, and uncomplicated malaria. Obeng‑Aboagye et al.^61^ showed no difference in MCP-1/CCL-2 between severe and uncomplicated *P. falciparum* malaria. Similarly, Cox-Singh et al.^63^ showed no significant increase in MCP-1/CCL-2 levels between complicated and non-complicated *P. knowlesi*-infected patients.

Six studies reported quantitative data on MCP-1/CCL-2 levels between participants with severe *Plasmodium* infections and those with non-severe malaria^38,39,46,56,60,62^. The meta-analysis included these studies to determine the pooled SMD of MCP-1/CCL-2 between two participant groups. Results revealed significantly higher MCP-1/CCL-2 levels in participants with severe *Plasmodium* infections compared to those with non-severe malaria (*P*: 0.04, SMD: 1.51, 95% CI: 0.06–2.95, *I*^*2*^: 98.5%, number of participants: 1371, Fig. 3).

**Fig. 3:**
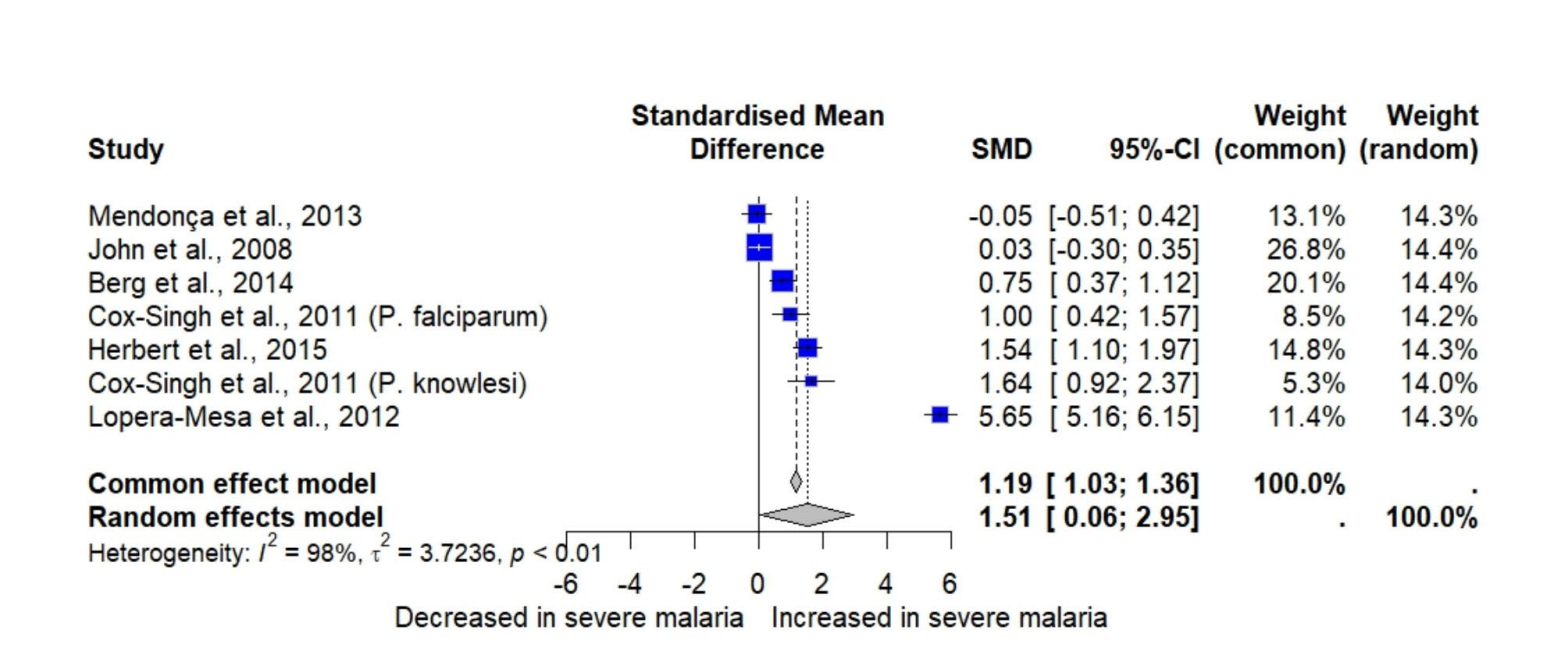
The forest plot illustrates the meta-analysis of MCP-1/CCL-2 levels between participants with severe *Plasmodium* infections and those with non-severe malaria. SMD represents the standardized mean differences. The CI illustrates the 95% confidence intervals from each study. The *I²* value of 98% reflects high heterogeneity across studies. The blue boxes illustrate an effect estimate from each study. The gray diamonds illustrate the pooled overall effect size.

The cumulative meta-analysis suggests a trend toward higher MCP-1/CCL-2 levels in severe *Plasmodium* infections compared to non-severe cases, though the result is not statistically significant (Supplementary Fig. 2). The subgroup meta-analysis of MCP-1/CCL-2 levels between participants with severe *Plasmodium* infections and those with non-severe malaria reveals substantial variability influenced by publication years, continents, and types of blood samples used for measuring MCP-1/CCL-2 levels (Table S4). The analysis based on publication years shows that studies from 2010 to 2019 report higher MCP-1/CCL-2 levels in severe infections. Geographically, studies from Africa and Asia exhibit increased MCP-1/CCL-2 levels in severe infections, although studies from Asia show no difference between the two participant groups. The analysis based on blood sample type demonstrated that studies using either plasma or serum showed no difference in MCP-1/CCL-2 levels between the two participant groups.

### Other findings about the alterations of MCP-1/CCL-2 in malaria

Frimpong et al.^54^ observed significantly higher MCP-1/CCL-2 levels in children with malaria compared to children with sepsis, but no difference in MCP-1/CCL-2 levels between children with sepsis and febrile controls. Cruz et al.^50^ showed that patients with malaria-HBV coinfection had elevated concentrations of MCP-1/CCL-2 compared to those with asymptomatic vivax malaria monoinfection, and coinfected individuals also had elevated MCP-1/CCL-2 levels compared to those with asymptomatic or symptomatic vivax malaria. Davenport et al.^64^ found no difference in MCP-1/CCL-2 levels between individuals with malaria alone and those exposed to both HIV-1 and *P. falciparum*.

Farrington et al.^37^ found that MCP-1/CCL-2 levels were elevated during acute infection in younger children but not in older children and that MCP-1/CCL-2 levels increased in children during an acute malaria episode regardless of parasite density at the time of infection. Han et al.^55^ revealed significantly higher MCP-1/CCL-2 levels in *P. vivax* compared to *P. falciparum* malaria. MacMullin et al., also observed significantly higher MCP-1/CCL-2 levels in *P. vivax* compared to *P. falciparum* malaria. Still, they found no difference in MCP-1/CCL-2 levels between *P. falciparum* and *P. ovale* malaria or between *P. vivax* and *P. ovale* malaria^34^. Fernandes et al.^53^ revealed no difference in MCP-1/CCL-2 levels between *P. falciparum* and *P. vivax* malaria. Cox-Singh et al., reported that patients with *P. knowlesi* malaria had significantly lower MCP-1/CCL-2 levels compared to those infected with *P. falciparum*. Also, MCP-1/CCL-2 levels decreased in patients with complicated *P. falciparum* but increased in patients with complicated *P. knowlesi* malaria^62^.

John et al.^38^ observed significantly higher MCP-1/CCL-2 levels in cerebral malaria patients who died compared to those who survived. Hojo‑Souza et al.^57^ found significantly lower MCP-1/CCL-2 levels in a *P. vivax*-treated group compared to *P. vivax* uncomplicated patients. Dieye et al.^51^ revealed that MCP-1/CCL-2 levels were significantly lower in cerebral malaria survivors compared to those who died. Royo et al.^65^ also showed no difference in MCP-1/CCL-2 levels between cerebral malaria patients who survived and those who died. Mendonça et al.^60^ found no difference in MCP-1/CCL-2 levels between asymptomatic malaria, symptomatic malaria, severe non-lethal malaria, and severe lethal malaria. Herbert et al.^56^ observed no difference in MCP-1/CCL-2 levels between severe non-cerebral malaria and multiorgan dysfunction. Armah et al.^45^ found no difference in MCP-1/CCL-2 levels between cerebral malaria and severe malarial anemia. Pappa et al.^40^ found statistically significant correlations between brain volume and MCP-1/CCL-2.

### Publication bias and sensitivity analysis

A test for funnel plot asymmetry could not be performed because the number of studies included in the meta-analysis was less than 10^66^. Sensitivity analyses were conducted to assess the robustness of the two meta-analyses in this study. For the meta-analysis of the difference in MCP-1/CCL-2 levels between *Plasmodium*-infected and *Plasmodium*-uninfected individuals, the results are robust but sensitive to including specific studies, particularly Dieye et al.^51^, which significantly influenced the overall effect size and heterogeneity. For the meta-analysis of the difference in MCP-1/CCL-2 levels between severe *Plasmodium*-infected and non-severe malaria, the influential analysis reveals that while some studies, particularly Lopera-Mesa et al.^39^, contribute significantly to the high heterogeneity, the overall effect remains statistically significant. This suggests that the findings are relatively robust.

## Discussion

MCP-1/CCL-2 is a chemokine that plays a major role in recruiting leukocytes, including monocytes, neutrophils, and lymphocytes, from the bloodstream across the vascular endothelium in response to infections or inflammations^67^. This chemokine-mediated recruitment is especially relevant in severe malaria, where inflammation can contribute to both parasite clearance and immunopathology^68^. The systematic review of MCP-1/CCL-2 levels in participants with *Plasmodium* infections revealed that most studies reported elevated MCP-1/CCL-2 levels in infected individuals compared to uninfected controls. However, the overall meta-analysis suggested no significant alteration in MCP-1/CCL-2 levels in *Plasmodium*-infected individuals, suggesting that the role of MCP-1/CCL-2 in malaria pathophysiology is complex. The high heterogeneity (*I²* = 97.2%) indicates substantial variability in effect sizes across studies, which limits the potential of using MCP-1/CCL-2 as a reliable diagnostic biomarker for malaria.

Subgroup analyses highlighted sources of heterogeneity likely due to differences in the continents, participant groups, *Plasmodium* species, and diagnostic methods for malaria. For different continents, the subgroup analysis showed that studies from Africa reported no significant difference in MCP-1/CCL-2 levels between infected and uninfected individuals, unlike studies from Asia and America. However, high heterogeneity was observed (*I*^*2*^ = 92.5%). This geographical variation may be due to the small number of studies included in the subgroup analysis, differences in participant groups, or the specific *Plasmodium* species involved. For example, studies in America^53,60^ and Asia^49,55,62^ enrolled participants infected with both *P. falciparum* and non-*P. falciparum* species, while African studies enrolled only participants infected with *P. falciparum*^37,51,64^. Varying levels of exposure to *Plasmodium* species may explain the differences in MCP-1/CCL-2 levels between African and Asian populations. In regions like sub-Saharan Africa, where *P. falciparum* transmission is endemic, individuals often develop partial immunity^69,70^, reducing the inflammatory response and potentially explaining the lack of significant MCP-1/CCL-2 elevation in these populations. In contrast, infections might trigger a more pronounced inflammatory response in regions like Asia, where malaria transmission is less frequent. This highlights the importance of considering the epidemiological and immunological background when interpreting the MCP-1/CCL-2 levels in patients with malaria. Nevertheless, even within a single study, the high levels of MCP-1/CCL-2 observed in infected Dogon children but not in infected Fulani children^47^ suggest that different participant groups (such as different age groups or pregnant women) may have varying immune responses to *Plasmodium* infections. Adults may have existing or acquired immunity from previous infections, leading to no difference between infected and uninfected participants. In contrast, children’s immune responses may not be fully developed due to their relative lack of exposure to infections^69^.

In the subgroup analysis, no significant difference in MCP-1/CCL-2 levels was observed between infected and uninfected pregnant women, suggesting that the role of MCP-1/CCL-2 during pregnancy may be complex. MCP-1/CCL-2 was reported to be related to monocyte infiltration into the intervillous space of malaria-infected placentas, suggesting that high MCP-1/CCL-2 expression may act as an early signal for monocyte recruitment^44^. Another study revealed that MCP-1 is crucial in recruiting immune cells into the *Plasmodium*-infected placenta^35^. MCP-1/CCL-2, in combination with MIP-1α and MIP-1β secreted from macrophages, acts as a chemoattractant for mononuclear cells in response to malarial hemozoin pigment^71^. In the subgroup analysis of children and adults, higher MCP-1/CCL-2 levels were observed in children infected with *Plasmodium* species compared to uninfected children. These results suggest that MCP-1/CCL-2 may play a distinct role in different participant groups, possibly reflecting varying immune responses to *Plasmodium* infections.

The observed differences in MCP-1/CCL-2 levels between malaria-infected and uninfected patients may also be influenced by the specific *Plasmodium* species involved. For instance, MCP-1/CCL-2 levels were reported to be lower in *P. knowlesi* infections compared to *P. falciparum*, and in *P. falciparum*, MCP-1/CCL-2 levels were associated with hemoglobin levels^62^. This suggests that the pathophysiological response, particularly macrophage activation, may differ between *Plasmodium* species, potentially contributing to variations in immune response and MCP-1/CCL-2 levels^62^. These differences could partially explain the variability in MCP-1/CCL-2 levels observed when comparing malaria-infected and uninfected patients. One study indicated significantly higher MCP-1/CCL-2 levels in patients infected with *P. vivax* compared to those with *P. falciparum*, suggesting that *P. vivax* may trigger a different inflammatory response^34^. While *P. vivax* can provoke inflammation and lead to a lower threshold for fever^72^, it is well-known that *P. falciparum* generally induces a stronger immune response due to the need to control its rapidly multiplying blood stages^73^. During *P. vivax* infection, high plasma levels of MCP-1/CCL-2 were detected regardless of parasite load, suggesting that these mediators are specifically induced by the infection^57^. In individuals with *P. vivax* infections and high parasitemia, a moderate to strong correlation was observed between IL-6, IL-10, and MCP-1, highlighting the importance of the IL-6/MCP-1/IFN-γ axis in regulating parasitemia in *P. vivax* infection^57^.

The meta-analysis examining differences in MCP-1/CCL-2 levels between participants with severe and non-severe malaria showed a statistically significant increase in MCP-1/CCL-2 levels in patients with severe malaria, with high heterogeneity (*I²* = 98%), indicating substantial variability among the included studies. Subgroup analyses revealed significantly higher MCP-1/CCL-2 levels in participants with severe *Plasmodium* infections compared to those with non-severe infections in the Asia subgroup but not in the Africa subgroup. These results suggest geography-related variability in MCP-1/CCL-2 response to the *Plasmodium* infection. Participants in Asia may have had less exposure to the infection during childhood, leading to a less developed immune response compared to those in sub-Saharan Africa, where the population is continuously exposed to *P. falciparum*^69^. This difference may explain why *Plasmodium* exposure in the Asian subgroup demonstrated a higher immune response and increased MCP-1/CCL-2 levels in severe-infected participants.

The high levels of MCP-1/CCL-2 observed in participants with severe malaria underscore the dual role of this chemokine. While MCP-1/CCL-2 is essential for mounting an effective immune response, excessive production can exacerbate inflammation and lead to complications such as cerebral malaria and acute renal failure^33^. This is particularly relevant in the context of cerebral malaria, where inflammation contributes to blood-brain barrier disruption, leading to severe neurological outcomes. As previously noted, MCP-1/CCL-2, in conjunction with other chemokines like MIP-1α, may be a key mediator of this disruption, as evidenced by its role in altering tight junction proteins and relate to increased blood-brain barrier permeability^56^. In addition, MCP-1/CCL-2, in conjunction with IL-8, is linked to changes in tight junction proteins, leading to increased permeability of brain endothelial cells^40^. For other severe complications, such as severe anemia, higher MCP-1/CCL-2 levels were positively correlated with parasitemia in patients with *P. falciparum* or *P. vivax* infections^53^. This may be explained by the increased production of MCP-1/CCL-2 in severe malaria, which is induced by the high levels of hemozoin, a byproduct of blood digestion in *Plasmodium* parasite infection during high parasite mass^74^.

Although MCP-1/CCL-2 is associated with severe malaria, a previous study demonstrated that MCP-1/CCL-2 is also linked to uncomplicated malaria, as asymptomatic malarial infection in co-infected patients exhibited higher MCP-1/CCL-2 levels than symptomatic *P. vivax* infections^50^. Additionally, another study found that MCP-1/CCL-2 levels are elevated in both uncomplicated and complicated malaria^38^, suggesting that MCP-1/CCL-2 may play a role in the host defense system during malaria, regardless of disease severity.

The systematic review and meta-analysis faced some limitations. First, the presence of publication bias cannot be ruled out, as the funnel plot asymmetry test was not conducted due to the limited number of studies. The lack of a formal publication bias analysis may affect the interpretation of our findings, as unpublished studies with negative or non-significant results might exist, potentially leading to an overestimation of the effect sizes. Second, the limited number of studies also affected the results of the subgroup analysis. Lastly, while the high heterogeneity in the meta-analysis presents challenges, it also reflects the diverse clinical and immunological landscapes of malaria. Factors such as parasite load, prior immunity, co-infections, and genetic predispositions all play a role in modulating the host’s immune response, and these must be carefully considered when interpreting the role of MCP-1/CCL-2 in malaria. Future studies should aim to integrate both clinical and immunological data to provide a more comprehensive understanding of how MCP-1/CCL-2 contributes to malaria pathogenesis and disease progression.

## Conclusion

The systematic review and meta-analysis suggest no statistically significant difference in MCP-1/CCL-2 levels in participants with *Plasmodium* infections overall. However, there was a significant increase in MCP-1/CCL-2 levels in patients with severe malaria. These findings suggest that MCP-1/CCL-2 may have potential as a prognostic biomarker for severe malaria. Future research should focus on large-scale, well-designed studies to validate the role of MCP-1/CCL-2 in malaria and further explore its prognostic potential.

## Electronic supplementary material

Below is the link to the electronic supplementary material.

Supplementary Material 1

Supplementary Material 2

Supplementary Material 3

Supplementary Material 4

Supplementary Material 5

## Data Availability

All relevant data are within the manuscript and its supporting information files.
