# Supplementary material for: A systematic review and meta-analysis of blood level of MCP-1/CCL-2 in severe and uncomplicated malaria
Source: Sci Rep. 2024 Nov 20;14:28738. doi: 10.1038/s41598-024-80201-y (PMC11579328; doi:10.1038/s41598-024-80201-y)
Supplement: Supplementary file 1 — Supplementary Material 1 [file 41598_2024_80201_MOESM1_ESM.docx]

**Table S1. Search terms**

**General keywords**

(“Chemokine CCL2” OR CCL2 OR “Monocyte Chemotactic and Activating Factor” OR “Monocyte Chemoattractant Protein-1” OR “Monocyte Chemoattractant Protein 1” OR “Chemokine (C-C Motif) Ligand 2” OR “CCL2 Chemokine” OR CCL2 OR “Monocyte Chemotactic Protein-1” OR “Monocyte Chemotactic Protein 1” OR MCP-1) AND (malaria OR plasmodium OR “Plasmodium Infection“ OR “Remittent Fever“ OR “Marsh Fever“ OR Paludism)

PubMed 4 July 2024

| No. | Key concept | Search terms | Results |
| --- | --- | --- | --- |
| 1. | Chemokine CCL2 | “Chemokine CCL2”[All Fields] OR CCL2[All Fields] OR “Monocyte Chemotactic and Activating Factor”[All Fields] OR “Monocyte Chemoattractant Protein-1”[All Fields] OR “Monocyte Chemoattractant Protein 1”[All Fields] OR “Chemokine (C-C Motif) Ligand 2”[All Fields] OR “CCL2 Chemokine”[All Fields] OR CCL2[All Fields] OR “Monocyte Chemotactic Protein-1”[All Fields] OR “Monocyte Chemotactic Protein 1”[All Fields] OR MCP-1[All Fields] OR “Chemokine CCL2”[MeSH Terms] OR CCL2[MeSH Terms] OR “Monocyte Chemotactic and Activating Factor”[MeSH Terms] OR “Monocyte Chemoattractant Protein-1”[MeSH Terms] OR “Monocyte Chemoattractant Protein 1”[MeSH Terms] OR “Chemokine (C-C Motif) Ligand 2”[MeSH Terms] OR “CCL2 Chemokine” [MeSH Terms] OR CCL2[MeSH Terms] OR “Monocyte Chemotactic Protein-1”[MeSH Terms] OR “Monocyte Chemotactic Protein 1”[MeSH Terms] OR MCP-1[MeSH Terms] | 38,459 |
| 2. | Malaria | "malaria"[MeSH Terms] OR "malaria"[All Fields] OR "malarias"[All Fields] OR "malaria s"[All Fields] OR "malariae"[All Fields] OR "plasmodium"[MeSH Terms] OR "plasmodium"[All Fields] OR "plasmodiums"[All Fields] OR "plasmodium s"[All Fields] OR "Plasmodium Infection"[All Fields] OR "Remittent Fever"[All Fields] OR "Marsh Fever"[All Fields] OR "malaria"[MeSH Terms] OR "malaria"[All Fields] OR "paludism"[All Fields] | 127,533 |
| 3. | #1 AND #2 | (“Chemokine CCL2”[All Fields] OR CCL2[All Fields] OR “Monocyte Chemotactic and Activating Factor”[All Fields] OR “Monocyte Chemoattractant Protein-1”[All Fields] OR “Monocyte Chemoattractant Protein 1”[All Fields] OR “Chemokine (C-C Motif) Ligand 2”[All Fields] OR “CCL2 Chemokine”[All Fields] OR CCL2[All Fields] OR “Monocyte Chemotactic Protein-1”[All Fields] OR “Monocyte Chemotactic Protein 1”[All Fields] OR MCP-1[All Fields] OR “Chemokine CCL2”[MeSH Terms] OR CCL2[MeSH Terms] OR “Monocyte Chemotactic and Activating Factor”[MeSH Terms] OR “Monocyte Chemoattractant Protein-1”[MeSH Terms] OR “Monocyte Chemoattractant Protein 1”[MeSH Terms] OR “Chemokine (C-C Motif) Ligand 2”[MeSH Terms] OR “CCL2 Chemokine” [MeSH Terms] OR CCL2[MeSH Terms] OR “Monocyte Chemotactic Protein-1”[MeSH Terms] OR “Monocyte Chemotactic Protein 1”[MeSH Terms] OR MCP-1[MeSH Terms]) AND ("malaria"[MeSH Terms] OR "malaria"[All Fields] OR "malarias"[All Fields] OR "malaria s"[All Fields] OR "malariae"[All Fields] OR "plasmodium"[MeSH Terms] OR "plasmodium"[All Fields] OR "plasmodiums"[All Fields] OR "plasmodium s"[All Fields] OR "Plasmodium Infection"[All Fields] OR "Remittent Fever"[All Fields] OR "Marsh Fever"[All Fields] OR "malaria"[MeSH Terms] OR "malaria"[All Fields] OR "paludism"[All Fields]) | 139 |

Embase 4 July 2024

| No. | Key concept | Search terms | Results |
| --- | --- | --- | --- |
| 1. | Chemokine CCL2 | “Chemokine CCL2”:ti,ab,kw,de OR CCL2:ti,ab,kw,de “Monocyte Chemotactic and Activating Factor”:ti,ab,kw,de OR “Monocyte Chemoattractant Protein-1”:ti,ab,kw,de OR “Monocyte Chemoattractant Protein 1”:ti,ab,kw,de OR “Chemokine (C-C Motif) Ligand 2”:ti,ab,kw,de OR “CCL2 Chemokine”:ti,ab,kw,de OR CCL2:ti,ab,kw,de OR “Monocyte Chemotactic Protein-1”:ti,ab,kw,de OR “Monocyte Chemotactic Protein 1”:ti,ab,kw,de OR MCP-1:ti,ab,kw,de OR “Chemokine CCL2”/exp OR CCL2/exp OR “Monocyte Chemotactic and Activating Factor”/exp OR “Monocyte Chemoattractant Protein-1”/exp OR “Monocyte Chemoattractant Protein 1”/exp OR “Chemokine (C-C Motif) Ligand 2”/exp OR “CCL2 Chemokine”/exp OR CCL2/exp OR “Monocyte Chemotactic Protein-1”/exp OR “Monocyte Chemotactic Protein 1”/exp OR MCP-1/exp | 86,984 |
| 2. | Malaria | malaria:ti,ab,kw,de OR plasmodium:ti,ab,kw,de OR ‘Remittent Fever’:ti,ab,kw,de OR ‘Marsh Fever’:ti,ab,kw,de OR Paludism:ti,ab,kw,de OR malaria/exp | 162,830 |
| 3. | #1 AND #2 | (RANTES:ti,ab,kw,de OR CCL5:ti,ab,kw,de OR “RANTES Protein”:ti,ab,kw,de OR “T-Cell RANTES Protein”:ti,ab,kw,de OR “CCL5 Chemokine”:ti,ab,kw,de OR RANTES/exp OR CCL5/exp OR “RANTES Protein”/exp OR “T-Cell RANTES Protein”/exp OR “CCL5 Chemokine”/exp) AND (malaria:ti,ab,kw,de OR plasmodium:ti,ab,kw,de OR ‘Remittent Fever’:ti,ab,kw,de OR ‘Marsh Fever’:ti,ab,kw,de OR Paludism:ti,ab,kw,de OR malaria/exp) | 381 |

Scopus 4 July 2024

| No. | Key concept | Search terms | Results |
| --- | --- | --- | --- |
| 1. | Chemokine CCL2 | TITLE-ABS-KEY ("chemokine ccl2" OR ccl2 OR "monocyte chemotactic and activating factor" OR "monocyte chemoattractant protein-1" OR "monocyte chemoattractant protein 1" OR "chemokine (c-c motif) ligand 2" OR "ccl2 chemokine" OR ccl2 OR "monocyte chemotactic protein-1" OR "monocyte chemotactic protein 1" OR mcp-1) | 69,682 |
| 2. | Malaria | TITLE-ABS-KEY (malaria OR plasmodium OR "plasmodium infection" OR "remittent fever" OR "marsh fever" OR paludism) | 164,231 |
| 3. | 1 AND 2 | (TITLE-ABS-KEY ("chemokine ccl2" OR ccl2 OR "monocyte chemotactic and activating factor" OR "monocyte chemoattractant protein-1" OR "monocyte chemoattractant protein 1" OR "chemokine (c-c motif) ligand 2" OR "ccl2 chemokine" OR ccl2 OR "monocyte chemotactic protein-1" OR "monocyte chemotactic protein 1" OR mcp-1)) AND (TITLE-ABS-KEY (malaria OR plasmodium OR "plasmodium infection" OR "remittent fever" OR "marsh fever" OR paludism)) | 335 |

MEDLINE 4 July 2024

| No. | Key concept | Search terms | Results |
| --- | --- | --- | --- |
| 1. | Chemokine CCL2 AND Malaria | (“Chemokine CCL2” OR CCL2 OR “Monocyte Chemotactic and Activating Factor” OR “Monocyte Chemoattractant Protein-1” OR “Monocyte Chemoattractant Protein 1” OR “Chemokine (C-C Motif) Ligand 2” OR “CCL2 Chemokine” OR CCL2 OR “Monocyte Chemotactic Protein-1” OR “Monocyte Chemotactic Protein 1” OR MCP-1) AND (malaria OR plasmodium OR “Plasmodium Infection“ OR “Remittent Fever“ OR “Marsh Fever“ OR Paludism) | 129 |

Ovid 4 July 2024

| No. | Key concept | Search terms | Results |
| --- | --- | --- | --- |
| 1. | Chemokine CCL2 AND Malaria | ("Chemokine CCL2" OR CCL2 OR "Monocyte Chemotactic and Activating Factor" OR "Monocyte Chemoattractant Protein-1" OR "Monocyte Chemoattractant Protein 1" OR "Chemokine (C-C Motif) Ligand 2" OR "CCL2 Chemokine" OR CCL2 OR "Monocyte Chemotactic Protein-1" OR "Monocyte Chemotactic Protein 1" OR MCP-1) AND (malaria OR plasmodium OR "Plasmodium Infection" OR "Remittent Fever" OR "Marsh Fever" OR Paludism) {No Related Terms}  Filter: limit to (ovid full text available and articles with abstracts and original articles) | 406 |

ProQuest 4 July 2024

| No. | Key concept | Search terms | Results |
| --- | --- | --- | --- |
| 1. | Chemokine CCL2 AND Malaria | ("Chemokine CCL2" OR CCL2 OR "Monocyte Chemotactic and Activating Factor" OR "Monocyte Chemoattractant Protein-1" OR "Monocyte Chemoattractant Protein 1" OR "Chemokine (C-C Motif) Ligand 2" OR "CCL2 Chemokine" OR CCL2 OR "Monocyte Chemotactic Protein-1" OR "Monocyte Chemotactic Protein 1" OR MCP-1) AND (malaria OR plasmodium OR "Plasmodium Infection" OR "Remittent Fever" OR "Marsh Fever" OR Paludism) | 714 |

Google Scholar 4 July 2024

| No. | Key concept | Search terms | Results |
| --- | --- | --- | --- |
| 1. | Chemokine CCL2 AND Malaria | (CCL2 OR MCP-1) AND (malaria OR plasmodium) | The first 200 articles |
