# Supplementary material for: A systematic review and meta-analysis of blood level of MCP-1/CCL-2 in severe and uncomplicated malaria
Source: Sci Rep. 2024 Nov 20;14:28738. doi: 10.1038/s41598-024-80201-y (PMC11579328; doi:10.1038/s41598-024-80201-y)
Supplement: Supplementary file 4 — Supplementary Material 4 [file 41598_2024_80201_MOESM4_ESM.docx]

**Table S4. Subgroup analysis of MCP-1/CCL-2 between groups of participants**

| ***Plasmodium*-infected versus *Plasmodium*-uninfected individuals** | | | | | | |
| --- | --- | --- | --- | --- | --- | --- |
| **Subgroup** | **Test for subgroup differences** | **SMD (95% CI)** | | | ***I^2^* (%)** | **Number of studies** |
| **Publication years** | 0.72 |  | | |  |  |
| 2000-2009 |  | 1.2114 [-1.3035; 3.7263] | | | 96.8 | 4 |
| 2010-2019 |  | 0.7145 [-0.3676; 1.7966] | | | 98.2 | 3 |
| **Study design** | 0.98 |  | | |  |  |
| Case-control study |  | 1.0260 [-2.7032; 4.7552] | | | 98.0 | 3 |
| Cross-sectional study |  | 1.0292 [-0.0968; 2.1552] | | | 96.0 | 2 |
| Cohort study |  | 0.9110 [ 0.7300; 1.0919] | | | 0.0 | 2 |
| **Continent** | < 0.0001 |  | | |  |  |
| Africa |  | 0.5599 [-0.3742; 1.4940] | | | 92.5 | 5 |
| Asia |  | 4.8205 [ 3.7954; 5.8456] | | | N/A | 1 |
| America |  | -0.2916 [-0.4732; -0.1099] | | | N/A | 1 |
| **Participants** | < 0.0001 |  | | |  |  |
| Pregnant women |  | 0.0274 [-1.2772; 1.3320] | | | 92.7 | 3 |
| Children |  | 1.0328 [ 0.7197; 1.3460] | | | N/A | 1 |
| Adults |  | 1.6106 [ 1.2476; 1.9736] | | | N/A | 1 |
| All age ranges |  | -0.2916 [-0.4732; -0.1099] | | | N/A | 1 |
| Not specified |  | 4.8205 [ 3.7954; 5.8456] | | | N/A | 1 |
| ***Plasmodium* species** | < 0.0001 |  | | |  |  |
| *P. falciparum* |  | 1.1330 [-0.7991; 3.0650] | | | 95.7 | 5 |
| *P. falciparum,* non*-P. falciparum* |  | 1.6106 [ 1.2476; 1.9736] | | | N/A | 1 |
| Non*-P. falciparum* |  | -0.2916 [-0.4732; -0.1099] | | | N/A | 1 |
| **Diagnostic method for malaria** | < 0.0001 |  | | |  |  |
| Microscopy |  | 1.1330 [-0.7991; 3.0650] | | | 95.7 | 5 |
| Microscopy/PCR |  | 1.6106 [ 1.2476; 1.9736] | | | N/A | 1 |
| Microscopy/RDT/PCR |  | -0.2916 [-0.4732; -0.1099] | | | N/A | 1 |
| **Methods for MCP-1/CCL-2** | 0.11 |  | | |  |  |
| Bead-based assay |  | 1.5579 [-0.0720; 3.1878] | | | 94.1 | 5 |
| ELISA |  | -0.4234 [-2.2446; 1.3978] | | | 98.0 | 2 |
| **Blood samples for MCP-1/CCL-2** | 0.96 |  |  |  |  |  |
| Plasma |  | 0.9865 [-0.6660; 2.6389] | | | 97.5 | 6 |
| Serum |  | 1.0328 [ 0.7197; 1.3460] | | | N/A | 1 |
| **Severe malaria versus non-severe malaria cases** | | | | | | |
| **Publication years** | 0.04 |  | | |  |  |
| 2000-2009 |  | 0.0261 [-0.2976; 0.3498] | | | N/A | 1 |
| 2010-2019 |  | 1.7554 [ 0.1475; 3.3633] | | | 98.5 | 5 |
| **Study design** | 0.64 |  | | |  |  |
| Case-control studies |  | 1.0167 [ 0.2453; 1.7880] | | | 89.4 | 3 |
| Cohort studies |  | 2.8372 [-2.6788; 8.3532] | | | 99.7 | 2 |
| Cross-sectional studies |  | 0.7470 [ 0.3733; 1.1207] | | | N/A | 1 |
| **Continent** | < 0.0001 |  | | |  |  |
| Africa |  | 2.1385 [-1.3238; 5.6007] | | | 99.4 | 3 |
| Asia |  | 1.3906 [ 1.0148; 1.7663] | | | 26.3 | 2 |
| America |  | -0.0457 [-0.5079; 0.4165] | | | N/A | 1 |
| **Age ranges** | 0.76 |  | | |  |  |
| Adults |  | 1.0518 [ 0.5687; 1.5349] | | | 57.0 | 2 |
| Children |  | 2.8372 [-2.6788; 8.3532] | | | 99.7 | 2 |
| All age ranges |  | 0.7491 [-0.8047; 2.3028] | | | 95.8 | 2 |
| ***Plasmodium* species** | 0.58 |  | | |  |  |
| *P. falciparum* |  | 2.0523 [-0.3783; 4.4828] | | | 99.1 | 4 |
| *P. falciparum*, non-*P. falciparum* |  | 0.7470 [ 0.3733; 1.1207] | | | N/A | 1 |
| Non-*P. falciparum* |  | 0.7748 [-0.8804; 2.4299] | | | 93.2 | 2 |
| **Diagnostic method for malaria** | 0.73 |  | | |  |  |
| Microscopy |  | 2.8372 [-2.6788; 8.3532] | | | 99.7 | 2 |
| Microscopy/PCR |  | 0.8348 [-0.1337; 1.8032] | | | 88.4 | 2 |
| Microscopy/RDT/PCR |  | 1.1353 [ 0.3585; 1.9120] | | | 86.4 | 2 |
| **Methods for MCP-1/CCL-2** | 0.79 |  | | |  |  |
| Bead-based assay |  | 1.5810 [-0.4869; 3.6488] | | | 99.0 | 5 |
| ELISA |  | 1.2807 [ 0.6529; 1.9085] | | | 46.4 | 1 |
| **Blood samples for MCP-1/CCL-2** | 0.001 |  | | |  |  |
| Plasma |  | 1.9717 [-0.5119; 4.4553] | | | 99.1 | 4 |
| Serum |  | 0.0261 [-0.2976; 0.3498] | | | N/A | 1 |
| Unclear |  | 1.2807 [ 0.6529; 1.9085] | | | 46.4 | 1 |

Abbreviations: ELISA, enzyme-linked immunosorbent assay; RDT, rapid diagnostic test; CI, confidence interval; SMD, standardized mean difference; PCR, polymerase chain reaction; N/A, not assessed.
