# Supplementary material for: A systematic review and meta-analysis of blood level of MCP-1/CCL-2 in severe and uncomplicated malaria
Source: Sci Rep. 2024 Nov 20;14:28738. doi: 10.1038/s41598-024-80201-y (PMC11579328; doi:10.1038/s41598-024-80201-y)
Supplement: Supplementary file 5 — Supplementary Material 5 [file 41598_2024_80201_MOESM5_ESM.pdf]

Supplementary Figures

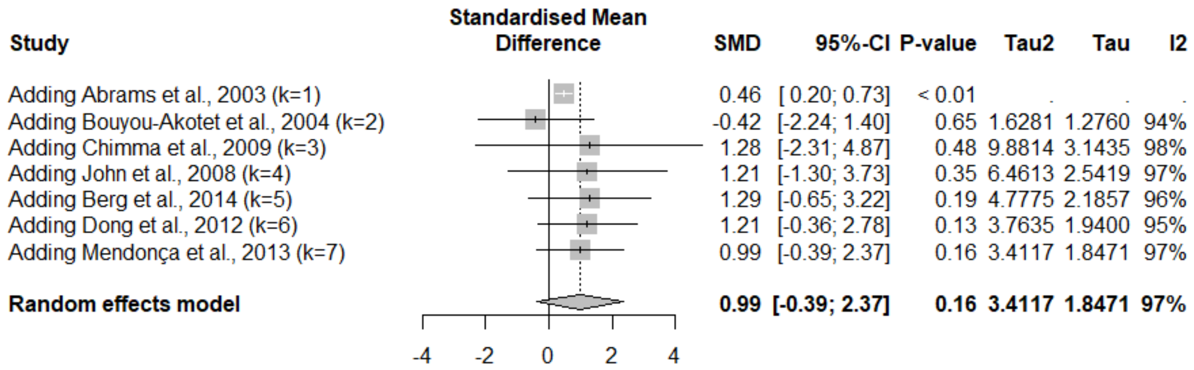

Supplementary Figure 1. The forest plot illustrates the cumulative meta-analysis of MCP-1/CCL-2 levels between *Plasmodium*-infected and uninfected individuals. SMD represents the standardized mean differences. The CI illustrates the 95% confidence intervals from each study. The I^2 value of 96% reflects high heterogeneity across studies. The gray boxes illustrate an effect estimate from each study. The gray diamond illustrates the pooled overall effect size. Individual studies are sequentially added, and their contributions to the overall effect are shown.

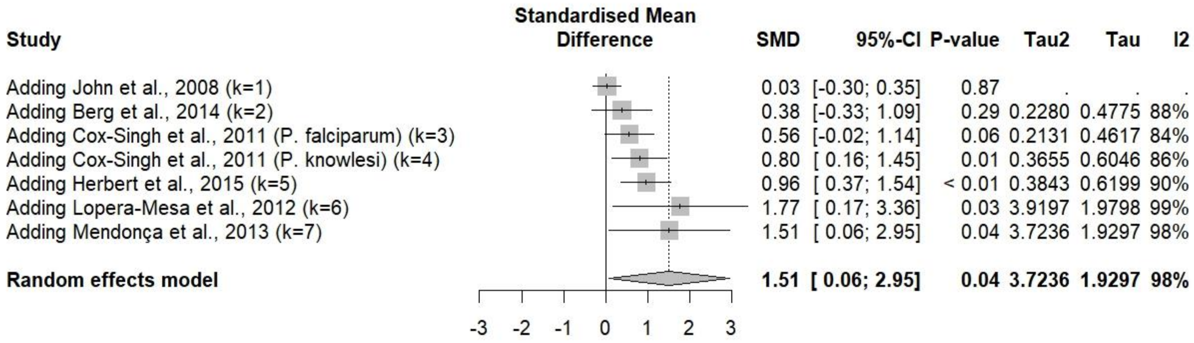

Supplementary Figure 2. The forest plot illustrates the cumulative meta-analysis of MCP-1/CCL-2 levels between participants with severe *Plasmodium* infections and those with non-severe malaria. SMD represents the standardized mean differences. The CI illustrates the 95% confidence

intervals from each study. The I^2 value of 99% reflects high heterogeneity across studies. The gray boxes illustrate an effect estimate from each study. The gray diamond illustrates the pooled overall effect size. Individual studies are sequentially added, and their contributions to the overall effect are shown.
